# Exploring the Mechanism of Dangguiliuhuang Decoction Against Hepatic Fibrosis by Network Pharmacology and Experimental Validation

**DOI:** 10.3389/fphar.2018.00187

**Published:** 2018-03-05

**Authors:** Hui Cao, Senlin Li, Rui Xie, Na Xu, Ying Qian, Hongdan Chen, Qinyu Hu, Yihong Quan, Zhihong Yu, Junjun Liu, Ming Xiang

**Affiliations:** ^1^School of Pharmacy, Tongji Medical College, Huazhong University of Science and Technology, Wuhan, China; ^2^Department of Traditional Chinese Medicine, The Central Hospital of Wuhan, Tongji Medical College, Huazhong University of Science and Technology, Wuhan, China

**Keywords:** Dangguiliuhuang decoction, hepatic fibrosis, network pharmacology, PPAR-γ, NF-κB

## Abstract

Dangguiliuhuang decoction (DGLHD) has been demonstrated to be effective in treating inflammatory, hepatic steatosis, and insulin resistance. In the study, we tried to elucidate the pharmacological efficacy and mechanism of DGLHD against liver fibrosis and predicate potential active ingredients and targets via network analysis and experimental validation. In the formula, we totally discovered 76 potential active ingredients like baicalein, berberine, and wogonin, and 286 corresponding targets including PTGS (prostaglandin-endoperoxide synthase) 2, PPAR (peroxisome proliferator-activated receptors) -γ, and NF-κB (nuclear factor-κB). Pathway and functional enrichment analysis of these putative targets indicated that DGLHD obviously influenced NF-κB and PPAR signaling pathway. Consistently, DGLHD downregulated levels of ALT (alanine transaminase) and AST (aspartate transaminase), reduced production of proinflammatory cytokines-TNF (tumor necrosis factor) -α and IL (Interleukin) -1β in serum and liver from mice with hepatic fibrosis, and inhibited hepatic stellate cell (HSC)-T6 cells proliferation. DGLHD decreased TGF (transforming growth factor) -β1 and α-SMA (smooth muscle actin) expression as well, maintained MMP (matrix metalloprotein) 13-TIMP (tissue inhibitor of metalloproteinases) 1 balance, leading to mitigated ECM (extracellular matrix) deposition *in vivo* and *in vitro*. Moreover, our experimental data confirmed that the alleviated inflammation and ECM accumulation were pertinent to NF-κB inhibition and PPAR-γ activation. Overall, our results suggest that DGLHD aims at multiply targets and impedes the progression of hepatic fibrosis by ameliorating abnormal inflammation and ECM deposition, thereby serving as a novel regimen for treating hepatic fibrosis in clinic.

## Introduction

Hepatic fibrosis is well recognized as a wound-healing response that occurs in liver following any type of acute or chronic injury, which is characterized by excessive accumulation of extracellular matrix (ECM) caused by both increased synthesis and deposition of newly formed components, and decreased or unbalanced degradation of ECM (Liu et al., 2006; Friedman, 2015). Perpetuation of the fibrotic reaction can lead to end-stage liver disease, cirrhosis, liver failure, and hepatocellular carcinoma, which represents a massive health care burden and increases globally (Hernandez-Gea and Friedman, 2011). However, few powerful antifibrotic drugs have been thoroughly validated in clinic or commercialized as a therapy (Ma et al., 2017). A limitation of the current antifibrotic approaches is that they are inefficiently taken up by activated hepatic stellate cells (HSCs) and may produce unwanted side effects, such as liver toxicity and promoting cancer development (Bataller and Brenner, 2005). Therefore, it is necessary for us to look for effective antifibrotic therapies.

Traditional Chinese medicine (TCM), as an important component of complementary and alternative medicine system, has been used to cure disease in China for over two thousand years (Tang et al., 2008). There are many TCM-based formulas and extracts of herbs that have been proved to be effective for alleviating liver fibrosis by suppressing inflammation, enhancing ECM degradation, and inhibiting HSCs activation, such as silymarin, emodin, tetrandrine, and ginkgo biloba extract (Xu and Lin, 2008; Federico et al., 2017).

Dangguiliuhuang decoction (DGLHD) is a classical TCM formula recorded in “Lan Shi Mi Cang,” which composed of angelica (AG), radix rehmanniae (RR), radix rehmanniae praeparata (RRP), scutellaria baicalensis (SB), coptis chinensis (CC), golden cypress (GC), and astragalus membranaceus (AM) (Cao et al., 2017), and is prepared in line with the China Pharmacopoeia standard of quality control. DGLHD has been used in clinic as alleviating diabetes, infection, myocarditis, and tuberculosis. Consistently, our previous studies have revealed that DGLHD possesses characteristics of anti-inflammation, immunosuppression, insulin sensitization, hypoglycemia, and hypolipidemia (Liu et al., 2015; Cao et al., 2017). In the formula, AG, RR, and RRP are the primary components and exert anti-inflammatory and hepatoprotective effects; GC, CC, and SB act as the ministerial components to strengthen the efficacy of primary agents; AM is the adjunctive component and used to enhance the action or reduce the side effects of primary and ministerial components. Additionally, DGLHD executes efficacy of nourishing Yin and clearing heat, and could be used to treat hepatic fibrosis with symptom of hot and humid. Increasing evidences have manifested that herbs in DGLHD, such as AM, AG, and CC, possess anti-inflammatory, anti-fibrotic, and hepatoprotective actions (Hong et al., 2013; Hua et al., 2014; Wu et al., 2016). However, whether or not DGLHD exerts protection for hepatic fibrosis and the potential mechanisms have not been elucidated.

Traditional Chinese medicine formula contained multi-component and multi-target, which is too complex to analyze by conventional experimental methods. Moreover, the clinical application of TCM in the world has been hampered because of ambiguous effects and mechanisms. It is thus necessary to clarify the scientific basis and the potential mechanisms of TCM applying new approaches. Network pharmacology, combined with pharmacology and pharmacodynamics, is a novel research field which clarifies the synergistic effects and the underlying mechanisms of numerous compounds by analyzing various networks of the complex and multi-levels interactions (Zhang et al., 2015). Since network description and analysis deliver a system-level understanding of drug action and disease complexity, the system-pharmacological model will be benefit for dissecting the functions of herbal medicines on special diseases (Zhang et al., 2014). Therefore, this study combined network pharmacology with experiments validation to clarify the underlying mechanism of DGLHD against hepatic fibrosis, in order to provide novel candidate for treating liver fibrosis.

## Materials and Methods

### Chemical Components of Each Herb in DGLHD

Chemical ingredients of each herb in DGLHD were obtained from TCM Database@Taiwan^1^ (Chen, 2011), traditional Chinese medicine systems pharmacology database (Ru et al., 2014) (TCMSP^2^), and associated literatures. Totally, we collected 545 compounds in DGLHD. Separately, there are 122 compounds in angelica (AG), 41 compounds in RR, 76 compounds in RRP, 141 compounds in SB, 132 compounds in GC, 48 compounds in coptis chinensis (CC), and 86 compounds in AM. The detailed information about chemical constituents of each herb in DGLHD was provided in Supplementary Table 1.

### Physicochemical Characteristics of Compounds in DGLHD

The molecular properties associated with pharmacology and chemical effects are mainly composed of molecular weight (MW), oral bioavailability (OB), drug-likeness (DL), the number of donor atoms for H-bonds (nHDon), the number of acceptor atoms for H-bonds (nHAcc) and Moriguchi octanol–water partition coefficient (logP) (MLogP). The characteristics of compounds in DGLHD were all obtained from TCMSP database and showed in Supplementary Table 2. We then analyzed the variables of basic properties in each herb of DGLHD.

### Predication of Potential Active Ingredients and Targets in DGLHD

Oral bioavailability represents the percentage of an orally administered dose of unchanged drug that reaches the systemic circulation, which reveals the convergence of the ADME process. High OB is often a key indicator to determine the drug-like property of bioactive molecules as therapeutic agents (Xu et al., 2012). DL is a qualitative concept used in drug design for an estimation on how “drug-like” a prospective compound is, which helps to optimize pharmacokinetic and pharmaceutical properties, such as solubility and chemical stability (Tao et al., 2013). In order to discover the active components of DGLHD, we selected ingredients meeting the requirements of both OB ≥ 30% and/or DL ≥ 0.1. Then we obtained putative targets of potential active ingredients in DGLHD from PubMed and TCMSP database, and removed those with no targets information.

### Related Targets of Hepatic Fibrosis

Liver fibrosis-associated targets were collected from the following resources. (1) Genecards^3^ (Safran et al., 2003), which is a searchable, integrative database that provides comprehensive, user-friendly information on all annotated and predicted human genes. And we collected 385 genes related to liver fibrosis from the database. (2) The Online Mendelian Inheritance in Man (OMIM) database^4^ (Hamosh et al., 2005). It is a comprehensive, authoritative compendium of human genes and genetic phenotypes that is freely available and updated daily. We searched the OMIM database with a keyword “hepatic fibrosis” and found 71 genes, such as PIK3CA, TLR4, TGFB1, NFKB1, TNF-α. (3) Kyoto Encyclopedia of Genes and Genomes (KEGG) Pathway Database^5^ (Kanehisa and Goto, 2000). We discovered 52 hepatic fibrosis-related targets appearing on the hepatic fibrosis pathway in the KEGG database. The detailed information about these targets were described in Supplementary Table 3. And we totally uncovered 458 targets linked with hepatic fibrosis after deleting redundancy.

### Pathway and Functional Enrichment Analysis

We performed Gene Ontology (GO) function enrichment analysis using Database for Annotation, Visualization and Integrated Discovery (DAVID^6^, version 6.8), which provides a comprehensive set of functional annotation tools for researchers to understand biological meaning behind large list of genes (Dennis et al., 2003). We also used pathway data obtained from KEGG database for pathway enrichment analysis.

### Network Construction

We established an interaction network among active ingredients of DGLHD, putative targets of DGLHD, and hepatic fibrosis-associated targets. The interaction network was visualized by Cytoscape 2.8.3 software^7^.

### Ethics Statement

Animal experiments in the study were approved by the Institutional Animal Care and Use Committee of Tongji Medical College, Huazhong University of Science and Technology, China (authorization number: No. 00263992). Twenty-four male C57BL/6J mice, 6 weeks of age, were purchased from Beijing Huafukang Bio-Technology Co. Ltd., and maintained at the experimental animal center of Tongji Medical College (Huazhong University of Science and Technology, China) with constant temperature 23 ± 2°C, a 12 h light/dark cycle, and free access to standard diet and water.

### DGLHD Preparation

Dangguiliuhuang decoction was provided by the Department of Traditional Chinese Medicine, the Central Hospital of Wuhan, Tongji Medical College, Huazhong University of Science and Technology, Wuhan, China. The final concentration of DGLHD was 0.13 g/ml. For biological studies, the 0.13 g/ml nominal concentration decoction was filtered (0.2 μm), sterilized, and diluted or concentrated.

### Cell Culture

Hepatic stellate cell-T6 cell line was purchased from Cell Center of Tongji Medical College and used for *in vitro* experimental validation. Cells were cultured in DMEM medium contained 10% heat-inactivated fetal bovine serum (FBS, Gibco, United States), 100 U/ml penicillin, and 100 μg/ml streptomycin at 37°C and 5% CO_2_.

In order to explore the effect and mechanism of DGLHD on activated HSC-T6 cells, quiescent HSC-T6 cells were stimulated by transforming growth factor (TGF)-β1 (5 ng/ml) with or without DGLHD (10 mg/mL) for 24 h. Then cells were collected for further detection.

### Cell Proliferation Assay

HSC-T6 cells were digested, counted, and seeded into 96-well plate at 3 × 10^3^ cells/plate. After 12 h, the cell medium was replaced with fresh medium included 5 ng/ml TGF-β1 in the presence of DGLHD or distilled water. At the end of 24 h incubation, cell proliferation was determined by MTT method at 490 nm.

### Animals and Treatment

Hepatic fibrosis was induced by intraperitoneally administration of 4% (v/v) carbon tetrachloride (CCl_4_) dissolved in olive oil (5 mL/kg body weight) twice a week for 6 weeks. Twenty-four mice were divided into three groups as follows: (1) Control: normal mice received distilled water; (2) Model: hepatic fibrosis mice received distilled water; (3) DGLHD: hepatic fibrosis mice received DGLHD (5 g/kg body weight). Eight mice were in each group and treated with DGLHD at the dosage according to the previous study (Cao et al., 2017). Distilled water and DGLHD were given orally by gavage at equal volumes for 6 weeks. Body weight of mice in all groups were monitored once a week for 6 weeks.

### Histomorphology Assay

Hepatic tissues were fixed in 10% formalin, embedded in paraffin, made into 5 μm thick sections, and stained with hematoxylin-eosin (HE) for histopathological examination or Masson trichrome dye for collagen formation. The histological sections were observed and photographed under an upright microscope. At least 10 independent fields per sample were assessed in each treatment group.

### Immunohistochemistry Analysis

α-SMA (smooth muscle actin) expression level in hepatic tissues was examined by immunohistochemistry. In brief, tissue sections were deparaffinized and eliminated endogenous peroxidase with 3% H_2_O_2_. Then the sections were blocked with 5% goat serum for 10 min, incubated with anti-rabbit α-SMA polyclonal antibody overnight, washed with PBS for three times, and incubated with HRP-conjugated anti-rabbit IgG for 30 min, followed by coloring with DBA reagent. Finally, the sections were evaluated by microscopy in a blinded manner.

### Serum Biochemical and Cytokine Analysis

Blood were obtained from mice, left 2 h at 4°C, centrifuged at 4°C, 6000 rmp for 10 min to collect the upper serum, and stored at -80°C. The concentrations of aspartate aminotransferase (AST) and alanine aminotransferase and (ALT) in serum were detected by commercial detection kits (Nanjing Jiancheng Bioengineering Institute, Nanjing, China) in accordance with the manufacturer’s protocols.

The concentrations of TGF-β1, tumor necrosis factor (TNF)-α, interferon (IFN)-γ, and interleukin (IL)-1β were evaluated by Cytometric Bead Array (CBA; BD Biosciences, San Jose, CA, United States) according to the manufacturer’s instructions. Briefly, 50 μL of mixed capture beads was mixed with 50 μL of the provided serum and 50 μL of the detected cytokines antibody, incubated in the dark for 3 h at room temperature. Then the samples were washed, centrifuged and resuspended in 300 μL of wash buffer. Finally, samples were analyzed using a BD C6 flow cytometer (BD Biosciences) and data was analyzed with BD FCAP Array software.

### Examination of ALT and AST in Liver

The hepatic tissues (15 mg) were homogenized by 150 μL ice-cold lysis buffer. Then tissue lysis liquid was centrifuged at 4°C, 10000 rpm for 15 min, the supernatant fraction was collected to measure the levels of ALT and AST in liver following the manufacturer’s instructions.

### Protein Analysis

Western blot analysis was conducted according to previous study (Cao et al., 2017). Generally, hepatic tissues and cells were lysed by RIPA buffer with 1% PMSF, and protein concentration was quantified with a BCA Protein Quantitation Kit (Thermo Fisher Scientific Inc., United States). Protein was separated by SDS-PAGE and transferred into PVDF membranes, blocked with 5% non-fat milk, and incubated with indicated antibodies overnight at 4°C. Proteins were determined with ECL chemiluminescence detection kit after incubation with secondary antibodies. Antibodies was used as follows: TGF-β1 (Abcam, ab92486), α-SMA (Proteintech, 14395-I-AP), PPAR (peroxisome proliferator-activated receptors)-γ (Cell Signaling Technology, 2435), NF-κB (nuclear factor-κB) (Signalway Antibody, 41228), β-actin (Proteintech, 60008-1-Ig). The amount of protein expression was unified by β -actin.

### Quantitative Real-Time PCR

Total RNA was isolated from HSC-T6 cells and livers using TRizol reagent according to the manufacturer’s instructions. RNA concentration and purity were examined by NanoDrop 2000 ultramicro spectrophotometer (Thermo Scientific, New York, NY, United States). 1 μg mRNA of each sample was reversely transcribed into cDNA using the Revert Aid First strand cDNA synthesis kit (Vazyme, Nanjing, China) and following the manufacturer’s instructions. qRT-PCR was performed using a SYBR Green Master Mix on CFX Connect Real-Time PCR System (Bioer, Hangzhou Bioer Technology Co., LTD.).

The qRT-PCR program included an initial denaturation step at 94°C for 1min, followed by 40 cycles of 94°C for 15 s, 60°C for 15 s, and 72°C for 30 s. β-actin was used as the internal control. Gene analysis was performed by the comparative cycle-threshold (Ct) (2^-ΔΔ*C*_t_^) method following normalization to the model group. Primer sequences are listed in Supplementary Table 4.

### Statistical Analysis

Data are shown as mean ± SEM. Statistical analysis was carried out using GraphPad Prism 5. Data between two groups were analyzed by Student’s *t*-test. Statistical analysis involved in more than two groups were performed using one-way ANOVA. Differences were considered statistically significant when *P*-value was less than 0.05.

## Results

### Ingredients Comparisons in DGLHD

**FIGURE 1:**
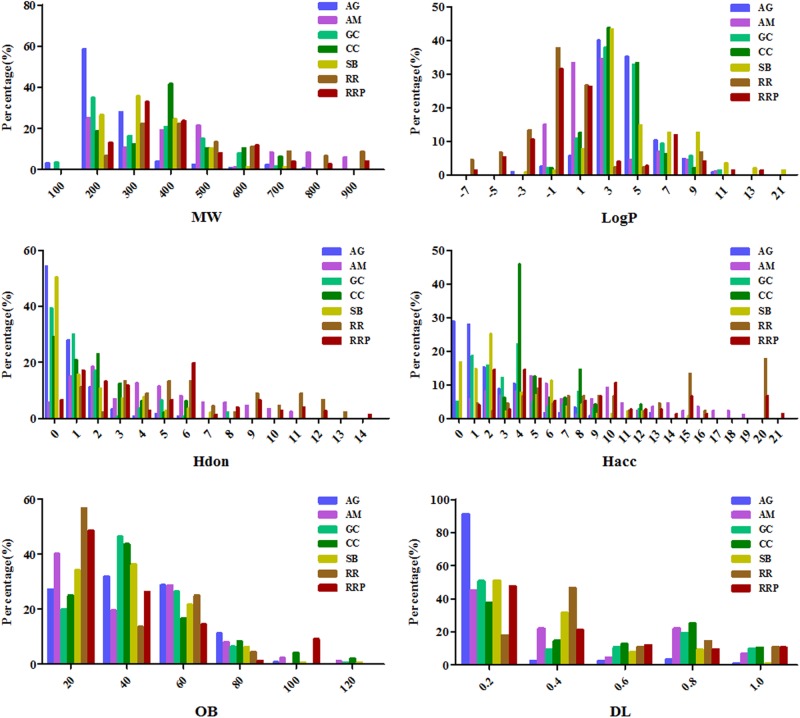
The profile distributions of all ingredients in Dangguiliuhuang decoction (DGLHD). There are seven herbs in DGLHD including angelica (AG), astragalus membranaceus (AM), golden cypress (GC), coptis chinensis (CC), scutellaria baicalensis (SB), radix rehmanniae (RR), radix rehmanniae praeparata (RRP). The molecular properties are mainly composed of molecular weight (MW), oral bioavailability (OB), drug-likeness (DL), the number of donor atoms for H-bonds (nHDon), the number of acceptor atoms for H-bonds (nHAcc) and Moriguchi octanol–water partition coefficient (logP) (MLogP).

To explore physicochemical similarities and differences of compounds in each herb of DGLHD, we compared their six properties: MW, MLogP, HDon, HAcc, OB, and DL (**Figure 1** and **Table 1**). For the primary herbs – AG, RR, RRP: AG is obviously distinguished from RR and RRP at the six aspects while the values of OB, DL, and MLogP in RR and RRP are similar; For the ministerial herbs – GC, CC, SB: the molecular characteristics of GC is alike with CC or SB, whereas CC is differ from SB in the aspects of MW, MLogP, HAcc, and DL. AM as the adjunctive herb is obviously different from other six herbs in MW, MLogP, HDon, and HAcc, whereas the values of OB and DL are almost analogous. In short, although there are various ingredients in the seven herbs of DGLHD, many of them still have similar chemical peculiarities.

**Table 1:** Comparison of ingredient properties among Dangguiliuhuang decoction (DGLHD).

	Primary ingredients			Ministerial ingredients			Adjunctive ingredient
			
Index	AG	RR	RRP	GC	CC	SB	AM
MW	203.18 ± 115.77^ΔΔ^	439.96 ± 209.05^∗∗^	361.52 ± 174.31^∗∗, #^	286.99 ± 141.05^ΔΔ^	342.19 ± 132.38^∗, Δ^	277.74 ± 106.74^##, ΔΔ^	418.03 ± 238.65
MLogP	3.26 ± 2.16^ΔΔ^	-1.10 ± 3.20^∗∗, ΔΔ^	0.06 ± 4.05^∗∗, ΔΔ^	3.10 ± 2.21^ΔΔ^	2.55 ± 1.87^Δ^	4.10 ± 3.35^∗∗, ##, ΔΔ^	1.47 ± 2.62
HDon	0.76 ± 1.12^ΔΔ^	6.18 ± 3.52^∗∗, ΔΔ^	4.60 ± 3.48^∗∗, #^	1.34 ± 1.75^ΔΔ^	1.77 ± 1.72^ΔΔ^	1.44 ± 1.93^ΔΔ^	4.16 ± 2.92
HAcc	2.00 ± 2.47^Δ^	10.47 ± 6.04^∗∗, ΔΔ^	7.88 ± 5.46^∗∗, #^	3.74 ± 2.75^ΔΔ^	5.50 ± 2.21^∗∗, ΔΔ^	3.48 ± 3.23^##, ΔΔ^	7.72 ± 4.81
OB	36.01 ± 19.39	24.87 ± 22.27^∗∗^	27.28 ± 25.45^∗∗^	34.51 ± 17.00	36.09 ± 23.55	31.42 ± 18.74	32.32 ± 23.70
DL	0.09 ± 0.16^ΔΔ^	0.41 ± 0.25^∗∗^	0.32 ± 0.27^∗∗^	0.33 ± 0.32	0.42 ± 0.31	0.23 ± 0.22^∗∗, ##, Δ^	0.31 ± 0.29


**AG, angelica; RR, radix rehmanniae; RRP, radix rehmanniae praeparata; SB, scutellaria baicalensis; CC, coptis chinensis; GC, golden cypress; AM, astragalus membranaceus.*^∗^P < 0.05, ^∗∗^P < 0.01 compared to AG or GC; ^#^P < 0.05, ^##^P < 0.01 compared to RR or CC; *^Δ^P < 0.05, ^ΔΔ^P < 0.01 compared to AM.**

### Potential Ingredients and Targets in DGLHD

As described above in the methods section, we discovered 76 potential active compounds in DGLHD after deleting redundancy (Supplementary Table 5), such as baicalein, berberine, quercetin, wogonin, and catapol, which was in line with our prior studies that these compounds are the major agents of DGLHD playing anti-inflammatory, immunosuppressive, anti-steatotic and anti-diabetic effects (Cao et al., 2017). Although ferulic acid had low DL value (DL = 0.06, OB = 54.97%), we selected it for further study based on its pharmacology effects. In other words, there are 5 compounds in AG, 2 compounds in RR, 11 compounds in RRP, 30 in compounds GC, 13 compounds in CC, 26 compounds in SB, and 11 compounds in AM.

Afterward, we collected 286 targets corresponding to active ingredients in DGLHD (Supplementary Table 5), including TNF-α, NF-κB, AP-1, PPAR-γ, PIK3CA, and MMPs, which were involved in inflammation, cell proliferation, or ECM deposition during the development of liver fibrosis. Then we selected those with degree values are more than 3 for further analysis (Supplementary Table 5).

### Network Construction and Analysis

**FIGURE 2:**
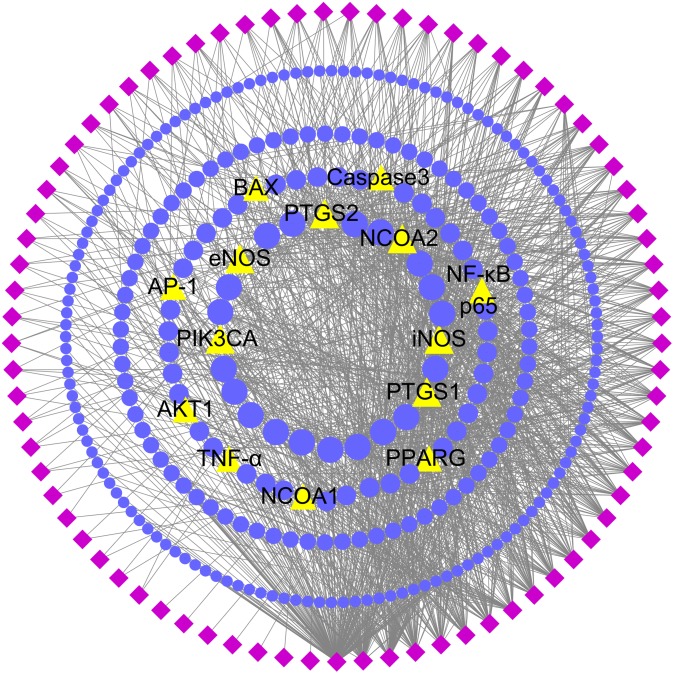
Potential active ingredient-target network of DGLHD acting on hepatic fibrosis. The network was based on the interaction among active compounds in DGLHD, potential targets of DGLHD, and targets associated with liver fibrosis. Diamond nodes represent the potential active ingredients of DGLHD; circular nodes represent the potential targets of DGLHD; triangle nodes represent the overlap between potential targets of DGLHD and hepatic fibrosis-associated targets. The size of these targets is positively related to their degrees in the network.

In order to comprehensively identify the mechanism of DGLHD treating hepatic fibrosis, we constructed a network among active ingredients, their correspondent targets, and liver fibrosis-related genes (**Figure 2**). In the network, targets in the inner circle are more closely linked with ingredients than those in the outer circle. PTGS (prostaglandin-endoperoxide synthase) 2, a key enzyme to regulate immune, inflammation, and cell proliferation, was influenced by 61 ingredients through TNF-α and NF-κB pathway. At the same time, PPAR-γ and NF-κBp65 were tuned by 12 and 7 ingredients via PPAR and AMPK signaling pathway, and TLR and TNF-α pathway, respectively (**Table 2**).

**Table 2:** The potential targets and their network degrees and related pathways.

Target	Description	Degree	Involved in pathway
PTGS2	Prostaglandin G/H synthase 2	61	TNF-α, NF-kappa B, VEGF signaling pathway
ADRB2	Beta-2 adrenergic receptor	24	cGMP-PKG, cAMP signaling pathway, Calcium
PIK3CA	Phosphatidylinositol-4,5-bisphosphate 3-kinase, catalytic subunit alpha	14	PI3K-Akt, RAS signaling pathway, hepatocellular carcinoma
PPARG	Peroxisome proliferator-activated receptor gamma	11	PPAR, AMPK, glucagon, Insulin signaling pathway
NF-κB p65	Nuclear factor-kappa B p65	7	TNF-α, Toll-like receptor, apoptosis, IL-17, HIF-1 signaling pathway, NAFLD, hepatitis B
Caspase3	Caspase3	7	Apoptosis – multiple species
Gsk3B	Glycogen synthase kinase 3 beta	6	Cell cycle, IL-17, B cell receptor signaling pathway, hepatitis C
BAX	BAX	6	Apoptosis, apoptosis – multiple species, NAFLD
AP-1	Nuclear transcription factor activation protein -1	5	TNF-α, toll-like receptor, IL-17 signaling pathway
TNF-α	Tumor necrosis factor	4	TNF-α, NF-κB, mTOR, MAPK signaling pathway
AKT1	RAC-alpha serine/threonine-protein kinase	4	PI3K-Akt, mTOR, FoxO, AMPK, VEGF signaling pathway


Additionally, there are 51 targets containing in both potential targets of DGLHD and liver fibrosis-associated targets, such as PTGS2, NF-κBp65, PPAR-γ, and PIK3CA (**Figure 2** and Supplementary Table 6), indicating the possible effects of DGLHD protecting against hepatic fibrosis. Pathway-enrichment analysis of these targets (**Table 2**) combined with their biological functions implied that these overlapped targets were in relation with the processes of inflammation, metabolism, and ECM deposition, resulting in a series of changes during the progression of hepatic fibrosis.

### Pathway and Functional Enrichment Analysis for Potential Targets of DGLHD

**FIGURE 3:**
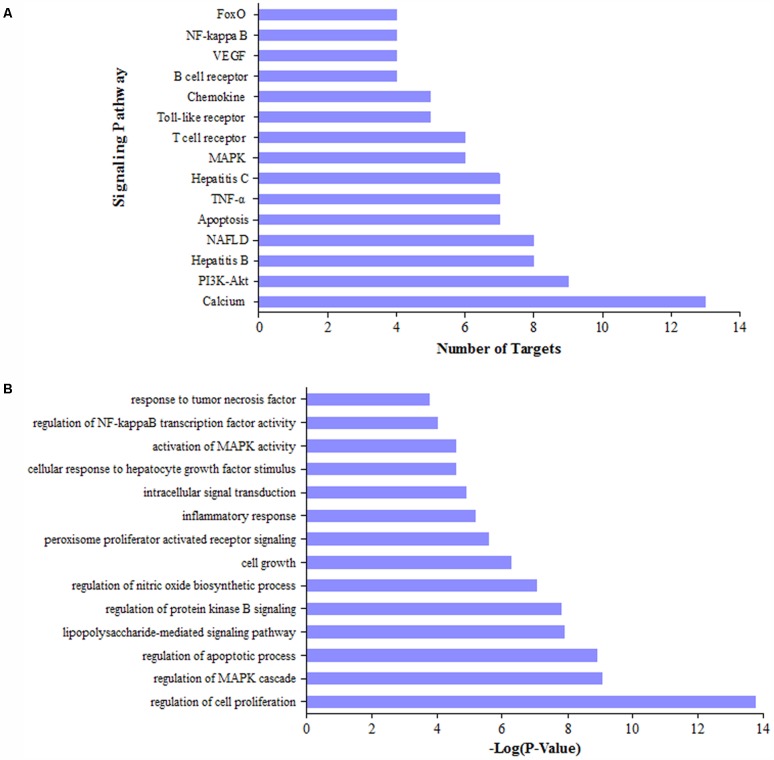
Pathway and function analysis for potential targets of DGLHD. We selected the targets of active ingredients in DGLHD with the degree values over 3, then performed KEGG pathway enrichment analysis **(A)** and GO function analysis **(B)**.

We collected 72 putative targets with the degree values over 3 from 286 potential targets. To identify relevant pathways and functions, we carried out pathway enrichment and Go function analysis for these putative targets. Results demonstrated that MAPK, NF-κB, and PI3K/Akt signaling pathway were obviously enriched except NAFLD (Non-alcoholic fatty liver disease) and hepatitis-related pathways (**Figure 3A**). Moreover, functional analysis data revealed that these putative targets not only modulated cell proliferation, apoptosis, growth, and inflammatory response but also tuned Akt, PPAR, and NF-κB activity (**Figure 3B**). It is important that PI3K/Akt, PPAR-γ, and NF-κBp65 could be regulated by multiple components in DGLHD (Supplementary Table 5). Therefore, these data provided theoretical evidences that DGLDH against liver fibrosis is possibly linked with the activity of PI3K/Akt, PPAR-γ, and NF-κBp65.

### DGLHD Alleviated Hepatic Fibrosis *in Vivo*

**FIGURE 4:**
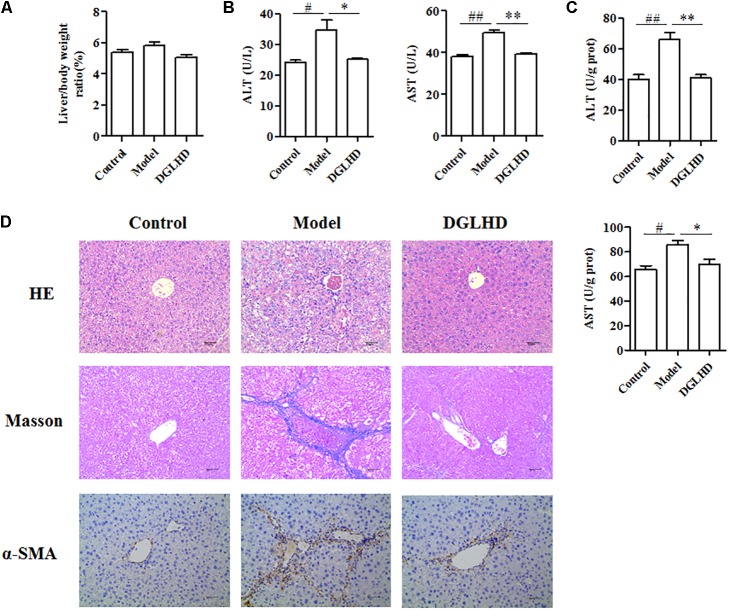
Dangguiliuhuang decoction ameliorated hepatic fibrosis in CCl_4_-induced fibrotic mice. C57BL/6J mice were administrated daily with water or DGLHD at 5.0 g/kg by intragastric gavage for 6 weeks (*n* = 8 for each group). **(A)** The ratio of liver weight to body weight. **(B)** Concentrations of ALT and AST in serum. **(C)** Concentrations of ALT and AST in liver homogenate. **(D)** Representative images of liver sections stained with hematoxylin-eosin or Masson or α-SMA antibody (magnification, 200×). Data are shown as mean ± SEM (*n* = 4–5). ^#^*P* < 0.05, ^##^*P* < 0.01 compared to Control. ^∗^*P* < 0.05, ^∗∗^*P* < 0.01 compared to Model.

In order to validate the anti-fibrotic effects of DGLHD, we established a mouse model of CCl_4_-induced liver fibrosis due to its convenient time frame. As shown in **Figures 4A–C**, treating fibrotic mice with DGLHD significantly decreased the levels of ALT and AST in serum and liver while had no obvious influence on the ratio of liver weight to body weight. We then assessed the pathomorphological changes of liver by HE and Masson staining. Results demonstrated that DGLHD treatment obviously diminished the accumulation of ECM and collagen, mitigated inflammatory infiltration, and contributed to maintain the normal morphology structure of liver tissue (**Figure 4D**).

### DGLHD Attenuated Inflammation of Hepatic Fibrosis Mice

**FIGURE 5:**
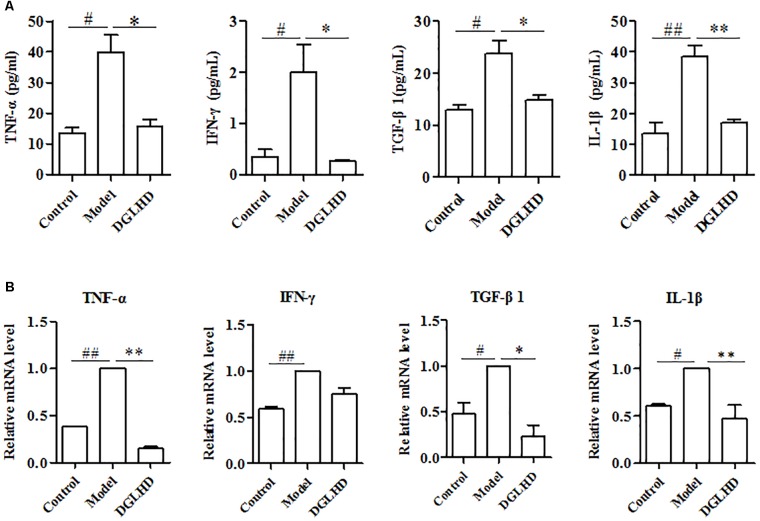
Dangguiliuhuang decoction diminished local and systemic inflammation in hepatic fibrosis mice. Liver and serum were separated from control and fibrotic mice given water or DGLHD (5.0 g/kg) at the indicated time. **(A)** Secretions of TNF-α, IFN-γ, TGF-β1, and IL-1β in serum. **(B)** mRNA expressions of TNF-α, IFN-γ, TGF-β1, and IL-1β were analyzed in liver by qRT-PCR. β-actin was used as the internal reference. Data are expressed as mean ± SEM (*n* = 4–5). ^#^*P* < 0.05, ^##^*P* < 0.01 compared to Control. ^∗^*P* < 0.05, ^∗∗^*P* < 0.01 compared to Model.

Considering inflammation is commonly related to progression of liver fibrogenesis (Lotersztajn et al., 2005), we thus examined the influence of DGLHD on local and systemic inflammation. Compared to control mice, the levels of TNF-α, IFN-γ, and IL-1β cytokines in serum as well as those genes in liver of fibrotic mice were all markedly increased. Interestingly, DGLHD treatment could reverse these elevated alteration (**Figure 5**).

Transforming growth factor-β1 is obviously overproduced by a variety of cells and is considered as a fibrogenic gene during hepatic fibrosis (Liu et al., 2006). Consistent with these findings, our results manifested that the levels of TGF-β1 in serum and liver from fibrotic mice were higher than that of control mice. And treatment with DGLHD notably inhibited these elevations (**Figure 5**). Overall, the improved hepatic fibrosis initiated by DGLHD could be attributed to alleviated proinflammatory environment.

### DGLHD Inhibited ECM Accumulation *in Vivo* and *in Vitro*

**FIGURE 6:**
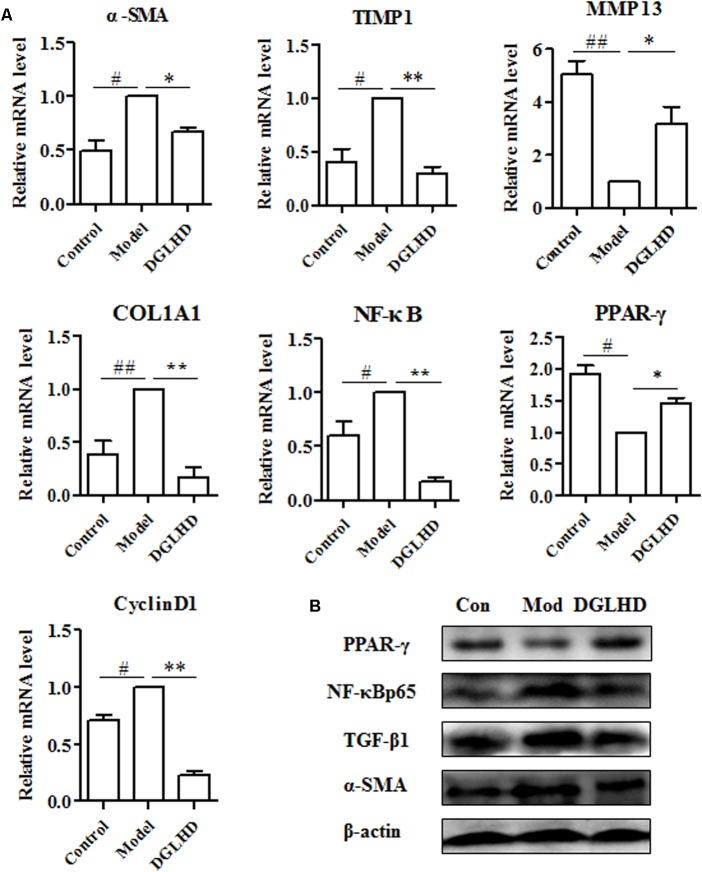
Dangguiliuhuang decoction suppressed ECM accumulation in liver. Hepatic tissues were isolated from normal mice and fibrotic mice treated with water or DGLHD (5.0 g/kg). **(A)** Fold change of gene expression was detected by qRT-PCR analyses. **(B)** Protein expressions of TGF-β1, α-SMA, PPAR-γ, NF-κB were determined by Western blotting. Proteins and genes were normalized to Model. β-actin was used as the internal reference. Data are presented as mean ± SEM (*n* = 4). ^#^*P* < 0.05, ^##^*P* < 0.01 compared to Control. ^∗^*P* < 0.05, ^∗∗^*P* < 0.01 compared to Model.

Given that liver fibrosis is characteristic of the imbalance of ECM synthesis and degradation, so we measured related gene expressions in liver. We observed DGLHD markedly elevated MMP13 mRNA expression, and diminished TIMP1, α-SMA, CoL1A1, and CyclinD1 gene levels (**Figure 6A**). And the protein levels of TGF-β1 and α-SMA were also apparently downregulated by DGLHD (**Figure 6B**). Immunohistochemical analysis also demonstrated that DGLHD attenuated α-SMA expression in hepatic tissues of fibrotic mice (**Figure 4D**).

**FIGURE 7:**
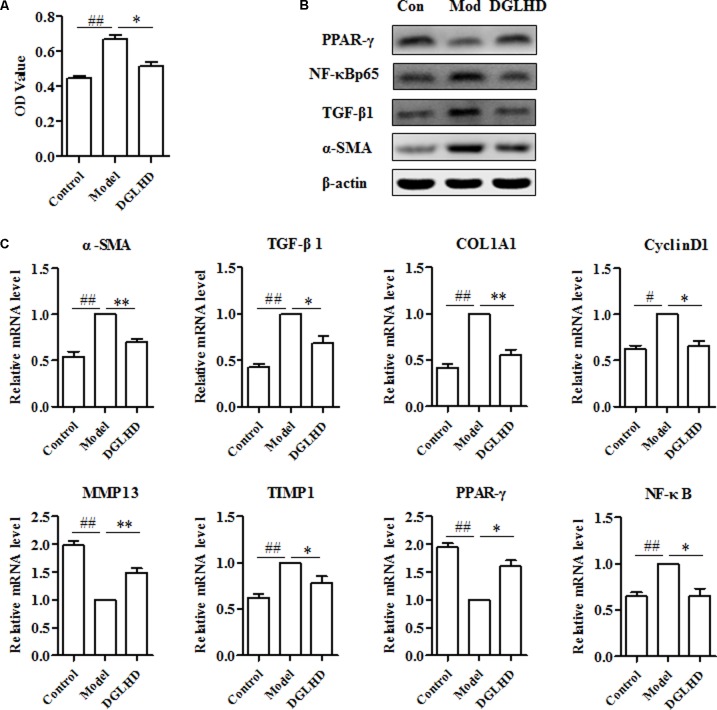
Dangguiliuhuang decoction exerted anti-fibrotic effects on HSC-T6 cells. HSC-T6 cells were activated by TGF-β1 (5 ng/mL) for 24 h to induce fibrosis. **(A)** Proliferation of HSC-T6 cells. **(B)** Western blot analysis of α-SMA, TGF-β1, PPAR-γ, and NF-κB protein expressions. **(C)** Expression level of fibrosis-related genes. Data shown as mean ± SEM (*n* = 4). ^#^*P* < 0.05, ^##^*P* < 0.01 compared to Control. ^∗^*P* < 0.05, ^∗∗^*P* < 0.01 compared to Model.

The key fibrogenic effector cell in liver is the activated HSCs, although other cells and processes can make significant contributions (Seki and Schwabe, 2015). Following liver injury, HSCs become activated, proliferate and produce ECM. Therefore, we examined whether DGLHD has direct anti-fibrotic effects on activated HSC-T6 cells. Results suggested that DGLHD restrained HSC-T6 cells proliferation and decreased CyclinD1 gene expression (**Figures 7A,C**). Gene analysis indicated DGLHD alleviated ECM production by reducing TIMP1 mRNA level and increasing MMP13 expression (**Figure 7B**). Further studies manifested the gene and protein expression levels of TGF-β1 and α-SMA in activated HSC-T6 cells were also diminished by DGLHD (**Figures 7B,C**). Taken together, these results provided evidences that DGLHD ameliorated hepatic fibrosis associated with amended balance between fibrogenesis and fibrolysis.

### DGLHD Exhibited Anti-fibrotic Effects via Modulating PPAR-γ and NF-κB

It has been reported that enhanced expression of PPAR-γ in activated HSCs inhibits collagen I expression, blocks TGF-β1 signaling, reduces proliferation, and increases cytoplasmic lipid droplets (Hernandez-Gea and Friedman, 2011). NF-κB also works as an important modulator of inflammation and fibrogenesis, and executes a major role in fibrosing liver disease (Luedde and Schwabe, 2011). As putative data described above, DGLHD plays positive effects on hepatic fibrosis probably through regulating PPAR-γ and NF-κBp65 molecules. Our experimental results further confirmed that DGLHD not only enhanced PPAR-γ gene and protein expression in fibrotic liver and activated HSC-T6 cells but also attenuated NF-κBp65 expression (**Figures 6**, **7**). Although network pharmacology data predicated that PTGS2 was modulated by 61 ingredients among 76 potential active compounds of DGLHD and PI3K/Akt signaling was significantly regulated by potential targets of DGLHD, we did not observe their alteration in fibrotic liver and activated HSC-T6 cells (data not shown). Collectively, DGLHD repressed hepatic fibrosis partially by modulating the expression of PPAR-γ and NF-κB molecules.

## Discussion

Hepatic fibrosis is a progressive disease that involves disruption of hepatic tissue architecture and accumulation of ECM in response to pathological insults, which is a common pathological process for the majority of liver diseases including end stage cirrhosis and hepatocellular carcinoma (Ellis and Mann, 2012). Currently, there are many different therapeutic targets being tested in antifibrotic drug development. They include: (1) to reduce the primary disease; (2) to reduce injury using hepatoprotectants; (3) to block myofibroblast activation, contractility and fibrogenesis; (4) to promote apoptosis or reversion of activated stellate cells, and (5) to stimulate matrix degradation (Friedman, 2015). However, most of treatments highlight the role of one cell, one cytokine, or one signaling molecule, without considering the complex process of hepatic fibrosis, thus cannot produce anticipated therapeutic effects. Thereby, it is urgent for us to uncover new antifibrotic therapies affecting two or more key pathogenic targets and/or pathways.

Dangguiliuhuang decoction, one of classical TCM, has multiple ingredients and targets. In this study, we analyzed the potential constituent components and targets of DGLHD using network pharmacology approach, and discovered 76 active compounds and 286 correspondent targets contained in DGLHD. Most of ingredients display anti-inflammatory and anti-fibrotic efficacies, such as berberine, baicalein, ferulic acid, catapol, coptisine, and wogonin (Xu et al., 2015; Li et al., 2016; Park, 2016; Wang N. et al., 2016; Chen et al., 2017; Lampiasi and Montana, 2017; Mahmoud et al., 2017; Shimizu et al., 2018). Ferulic acid, baicalein, coptisine, and wogonin downregulate NF-κB signaling (Chen et al., 2017; Lampiasi and Montana, 2017; Shimizu et al., 2018), while berberine, catapol, and quercetin modulate PPAR-γ and NF-κB activities (Le et al., 2015; Chuang et al., 2016; Li et al., 2016; Park, 2016; Wang Y.C. et al., 2016; Lee et al., 2017; Mahmoud et al., 2017). Additionally, pathway and functional enrichment analysis for putative targets revealed that DGLHD mainly regulated TNF-α, NF-κB, PPAR, and PI3K/Akt signaling pathway, which were all involved in fibrogenesis (Seki and Schwabe, 2015). In line with these findings, experimental results in the study demonstrated that DGLHD not only weakened inflammation of fibrotic mice, but also directly inhibited HSC activation and ECM deposition, eventually restraining the progression of liver fibrosis.

Following liver injured by CCl_4_ or other factors, various cells will produce proinflammatory cytokines, such as TNF-α and IL-1β, that contribute to the development of fibrosis (Hernandez-Gea and Friedman, 2011). Here, we found that treatment with DGLHD downregulated levels of TNF-α and IL-1β in serum and liver of fibrotic mice. Of note, the level of IFN-γ in serum and liver was reduced by DGLHD as well. Although it has been reported that IFN-γ exerts direct antifibrogenic effects in HSCs. (Jeong et al., 2011), we speculate that the IFN-γ secretion recovered to normal upon hepatic fibrosis was significantly ameliorated by DGLHD.

Moreover, fibrotic pathologies are associated with increased level of TGF-β1 that initially recruits inflammatory cells and fibroblasts into an area of injury and then stimulates these cells to produce cytokines and ECM (Liu et al., 2006). And TGF-β1 overexpression in transgenic animals induces spontaneous liver fibrosis (Kisseleva and Brenner, 2008). In this study, we observed DGLHD treatment not only attenuated TGF-β1 production in serum and liver of fibrotic mice, but also directly suppressed TGF-β1 protein and gene expressions in activated HSC-T6 cells. Favorably, these results verified our targets prediction that hepatic fibrogenesis alleviated by DGLHD is associated with regulation of TGF-β1 expression. Collectively, it seems likely that improved fibrosis caused by DGLHD is related to attenuated inflammation and ECM accumulation with lowered TGF-β1 activity.

Various inflammatory cytokines like IL-1β and TNF-α activate NF-κB, a transcription factor that acts as a key regulator of inflammation and cell death, thus exerting a major role in chronic liver disease (Seki and Schwabe, 2015). Selective inhibition of NF-κB by using the NF-κB decoy nucleotides inhibits CCl_4_-induced liver inflammation and fibrosis (Son et al., 2007). In addition, NF-κB activation in HSCs promotes fibrogenesis from increased HSC survival and activation. To our excitement, experimental data revealed that DGLHD diminished protein and mRNA levels of NF-κB *in vivo* and *in vitro*, which was in consistent with the results of pathway and functional enrichment analysis that DGLHD obviously affected NF-κB signaling pathway and NF-κB transcriptional activity. Importantly, baicalein, berberine, ferulic acid, catapol, coptisine and quercetin contained in DGLHD have been reported to exert anti-inflammatory and/or anti-fibrotic effects via inhibition of NF-κB pathway (Sun et al., 2010; Le et al., 2015; Xu et al., 2015; Wang Y.C. et al., 2016; Chen et al., 2017; Lampiasi and Montana, 2017; Lee et al., 2017; Shimizu et al., 2018). Thereby, it seems reasonable that DGLHD repressed the development of fibrogenesis by modulating NF-κB activation.

It is well known that MMP13 and TIMP1 are mainly expressed by HSC cells and influenced by cytokines like IL-1β, TNF-α, and TGF-β1 (Hernandez-Gea and Friedman, 2011). Production of MMPs and TIMPs is tightly regulated according to the activation state of HSC. In chronic liver injury, differently regulated MMP13 and TIMP1 expression leads to a positive feedback loop with subsequent fibrogenesis (Hemmann et al., 2007). In activated HSCs especially the expression of TIMP-1 is upregulated leading to the inhibition of MMP activity and subsequent accumulation of matrix proteins (Hemmann et al., 2007). Our *in vivo* and *in vitro* experimental results indicated that DGLHD decreased TIMP1 expression while increased MMP13 expression, and restored MMP13-TIMP1 balance. Additionally, DGLHD treatment also apparently decreased the levels of α-SMA and COL1A1, which were recognized as mesenchymal markers (Kisseleva and Brenner, 2008). Hence, DGLHD improved liver fibrosis is linked with mitigated ECM accumulation.

It has been reported that PPAR-γ expressed in HSCs plays a crucial role in the maintenance of quiescent HSCs (Hazra et al., 2004). Stimulation of PPAR-γ blocks TGF-β1/Smad signaling, inhibits HSC proliferation and induces apoptosis in *vitro* and in *vivo* (Hazra et al., 2004; Lin and Chen, 2008). Our network data implied that PPAR-γ was modulated by several potential active ingredients like berberine, catalpol, quercetin, which have been demonstrated to prevent liver injury, HSCs activation and inflammation through upregulating PPAR-γ (Chuang et al., 2016; Li et al., 2016; Park, 2016; Mahmoud et al., 2017). Additionally, pathway-enrichment analysis also revealed that PPAR signaling pathway was notably enriched by potential targets of DGLHD. Excitingly, our validate experiments showed that DGLHD enhanced PPAR-γ activity in fibrotic liver as well as HSC-T6 cells. Therefore, ameliorated inflammation and collagen deposition generated by DGLHD could be at least partly attributed to elevated PPAR-γ activation.

## Conclusion

**FIGURE 8:**
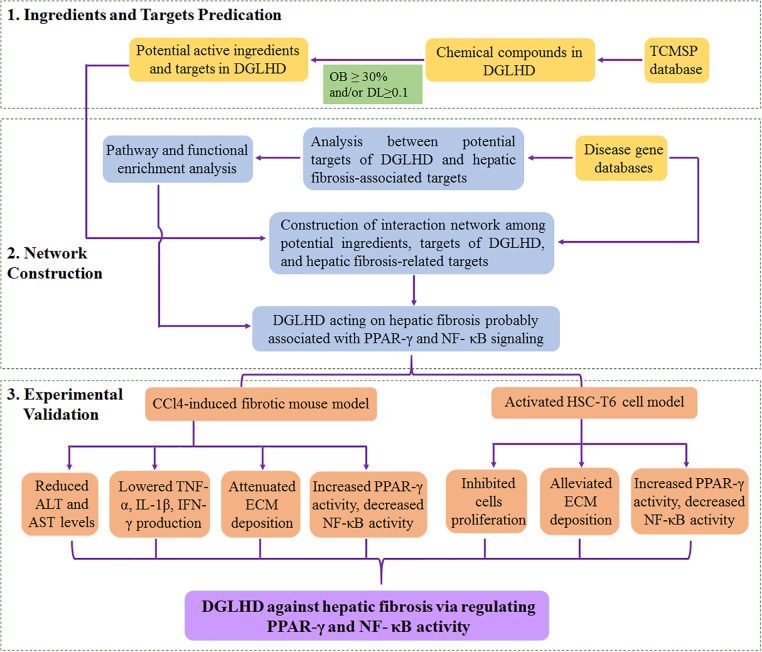
The overview of systematic strategies for discovering the mechanisms of DGLHD acting on hepatic fibrosis.

We predicted that DGLHD exerted protection against hepatic fibrosis partially through regulating NF-κB and PPAR-γ signaling via an integrative analysis approach combining potential active ingredients, putative targets of compounds in herbal formula, and targets of a specific disease. Further we verified with experimental evidence that DGLHD improved liver fibrogenesis by suppressing inflammatory status and recovering balance of ECM synthesis and degradation through modulating the activity of NF-κB and PPAR-γ (**Figure 8**). The research provided new treatment regimens for fibrotic patients according to the paradigm of “multi-component, multi-target, multi-pathway.” However, the precise active ingredients and corresponding targets in DGLHD need to be further investigated.

## Author Contributions

HC drafted the manuscript and analyzed the data. HC and SL performed the experiments and prepared the figures and tables. RX, NX, YQ, HDC, and QYH performed the experiments. YHQ and ZHY provided suggestions and material support. JJL and MX designed the study and provided database support. MX obtained the funding, supervised the whole project and reviewed the manuscript.

## Conflict of Interest Statement

The authors declare that the research was conducted in the absence of any commercial or financial relationships that could be construed as a potential conflict of interest.

## Footnotes

1 http://tcm.cmu.edu.tw/zh-tw/

2 http://lsp.nwu.edu.cn/tcmsp.php

3 http://www.genecards.org

4 http://www.omim.org/

5 http://www.genome.jp/kegg/

6 https://david.ncifcrf.gov/

7 http://www.cytoscape.org
