# Supplementary material for: Exploring the Mechanism of Dangguiliuhuang Decoction Against Hepatic Fibrosis by Network Pharmacology and Experimental Validation
Source: Front Pharmacol. 2018 Mar 5;9:187. doi: 10.3389/fphar.2018.00187 (PMC5844928; doi:10.3389/fphar.2018.00187)
Supplement: Supplementary file 1 [file Table_1.docx]

**Supporting Information**

**Exploring the Mechanism of Dangguiliuhuang Decoction against Hepatic Fibrosis by Network Pharmacology and Experimental Validation**

Hui Cao^1#^, Senlin Li^1#^, Rui Xie^1^, Na Xu^1^, Ying Qian^1^, Hongdan Chen^1^, Qinyu Hu^1^, Yihong Quan^2^, Zhihong Yu^2^, Junjun Liu^1^*, Ming Xiang^1^*

^1^School of Pharmacy, Tongji Medical College, Huazhong University of Science and Technology, Wuhan, China

^2^Department of Traditional Chinese Medicine, The Central Hospital of Wuhan, Tongji Medical College, Huazhong University of Science and Technology, Wuhan, China

***Correspondence:**

Ming Xiang, E-mail: xiangming@tjmu.edu.cn

Junjun Liu, E-mail: junjun.liu@hust.edu.cn

^#^These authors contributed equally to this work.

**Table S1 Ingredients contained in DGLHD**

**Table S2 Physicochemical properties of compounds in DGLHD**

**Table S3 Hepatic fibrosis-related targets**

**Table S4 Primer sequences applied to quantitative real-time PCR analysis**

**Table S5 Potential active ingredients and targets in DGLHD**

**Table S6 Overlapped targets between potential targets of DGLHD and hepatic fibrosis-related targets**
